# Dengue Virus Infection Is through a Cooperative Interaction between a Mannose Receptor and CLEC5A on Macrophage as a Multivalent Hetero-Complex

**DOI:** 10.1371/journal.pone.0166474

**Published:** 2016-11-10

**Authors:** Yen-Lung Lo, Gunn-Guang Liou, Jia-Huei Lyu, Michael Hsiao, Tsui-Ling Hsu, Chi-Huey Wong

**Affiliations:** 1 Genomics Research Center, Academia Sinica, Taipei, Taiwan; 2 Institute of Molecular Biology, Academia Sinica, Taipei, Taiwan; 3 Chemical Biology and Molecular Biophysics, Taiwan International Graduate Program, Academia Sinica, Taipei, Taiwan; 4 Institute of Biochemical Sciences, National Taiwan University, Taipei, Taiwan; Deutsches Primatenzentrum GmbH—Leibniz-Institut fur Primatenforschung, GERMANY

## Abstract

Dengue fever is a mosquito-borne viral pandemic disease that is widespread in the tropical and subtropical areas. Dengue virus uses human mannose-binding receptor (MR) and DC-SIGN on macrophages as primary receptors, and CLEC5A as signaling receptor to sense the dengue virus invasion and then to signal and stimulate macrophages to secrete cytokines. But the interplay between MR/DC-SIGN and CLEC5A is unknown. Here we demonstrate a plausible mechanism for the interaction, i.e. MR/DC-SIGN first attracts the virus with high avidity, and the virus concurrently interacts with CLEC5A in close proximity to form a multivalent hetero-complex and facilitate CLEC5A-mediated signal transduction. Our study suggests that the cooperation between a high-avidity lectin-virus interaction and a nearby low-avidity signaling receptor provides a necessary connection between binding and signaling. Understanding this mechanism may lead to the development of a new antiviral strategy.

## Introduction

Dengue fever is a mosquito-borne viral pandemic disease that is widespread in the tropical and subtropical areas, putting more than 4 billion people at risk, with 390 million infections and 96 million cases of illness annually[1, 2]. Understanding the mechanisms of dengue virus and host interaction, an important step in virus-mediated pathogenesis and disease development[3], is vital for the development of antiviral strategies.

Dengue infection starts with the attachment of the virus to the host cell surface, and then the virus diffuses along the cell surface to search for receptors on the host cell [4]. The host cell is often a human macrophage where dengue virus uses human mannose-binding receptor (MR) and dendritic cell-specific intercellular adhesion molecule-3-grabbing non-integrin (DC-SIGN) as major receptors to stabilize the virus from surface diffusion and invade the host cell by triggering endocytosis[5, 6]. After virus uptake, macrophages, important players in the innate immune system, also use MR and DC-SIGN to present the digested antigens on the cell surface for the subsequent activation and antibody production[3]. This mechanism is used by the virus to invade cells and is also the virus antigen presenting machinery that defends the host from further infection [7]. On the other hand, macrophages use the virus pattern recognition receptor, C-type lectin domain family 5 member A (CLEC5A) instead of MR and DC-SIGN to sense dengue virus invasion and then stimulate macrophages to secrete cytokines to trigger a host cytokine-dependent immune response[8–11]. MR/DC-SIGN and CLEC5A on the macrophage cell surface contribute not only to anti-virus antibody production but also to other cytokine-dependent immune responses. However, how these two kinds of receptors interact with the virus and allow viral entry and infection are not known [12–14].

In this study, we demonstrate that the interaction between dengue virus and CLEC5A is relatively weak, with dissociation constant (*K*_D_) in the micromolar range. As MR or DC-SIGN binds the virus with *K*_D_ in the sub-nanomolar range, CLEC5A is unable to compete with MR and DC-SIGN in binding to the virus [5, 8, 15]. With the use of digital image correlation analysis, we further investigate the localization of CLEC5A and MR/DC-SIGN on dengue virus-infected macrophages. Using an immunogold-assisted transmission electron microscope (TEM), we observed that both CLEC5A and MR/DC-SIGN are located in a small area. These results provide a plausible mechanism that CLEC5A-mediated dengue virus invasion does not necessarily compete with dengue virus binding to MR and DC-SIGN. With the high binding avidity of MR and DC-SIGN to dengue virus in a confined area, the nearby CLEC5A can concurrently gain a good contact with the virus and transduce the signal. This kind of cooperative interaction between multiple receptors and virus is further demonstrated in this study resulting in a better understanding of dengue virus infection and a new direction for developing anti-dengue strategies.

## Materials and Methods

### Ethics statement

The study was carried out with the approval of the Institutional Review Boards on Biomedical Science Research, Academia Sinica and with the permission from the ethics committees of the Academia Sinica (AS-IRB-BM-102033). No informed consent was required because the blood samples were received from anonymous donors and approved by the ethics committees. The anonymous donors in this study are defined as blood bank (Taiwan Blood Service Foundation) donors who signed the consent form and agreed to give blood for storage at the blood bank as part of the collection for transfusion to non-specified recipients and for use by scientific research. The donors agreed to remove their personal information before transferring the blood to its final destination.

### Bio-layer interferometry for measuring receptor-virus binding kinetics

Bio-layer interferometry (BLI, ForteBio) is used for the kinetic study of human receptor-Fc conjugates interacting with dengue virus. The BLI method uses a tip of fiber-optic biosensor coated with a proprietary matrix and a receptor of interest. When the biosensor tip is dipped into a sample in a 96-well microplate containing the target molecules that bind to the receptor on the tip, the tip forms a molecular layer, and its thickness increases as more target molecules bind to the surface. The thickness change is detected as wavelength shift or spectral shift (nm) due to the nature of interferometry. This spectral shift is monitored by the detector, and reported as the sensorgram according to the change in nm shift. Monitoring the interference pattern in real time provides kinetic data of molecular interactions [16, 17].

Fc-CLEC5A and Fc-DC-SIGN were prepared as previously described [18, 19]. Fc-MR (Fc-CRD4-7) is a gift from Dr. Simon Gordan and Dr. Luisa Martinez-Pomares[5]. Dengue virus is a gift from Dr. Yi-Ling Lin, IBMS, Academia Sinica, and is prepared as previously described [20, 21]. The biosensor (ForteBio) is immobilized with the Fc-dengue receptor conjugates, and react with dengue virus in Tyrode's solution (NaCl(135.6mM), KCl(5.4), CaCl2(1.8), MgCl2(0.53), Glucose(5.56), Hepes(5), pH 7.4, 300mOsm) at room temperature. The concentration of dengue virus in the study was determined based on the equivalent of dengue virus envelope protein. Data were analyzed with 1:1 binding model as there is no other proper binding model to be fitted with this type of multivalent binding.

### Cell culture, antibodies, immunoprecipitation assays and virus handling

Monocytes were isolated to high purity by magnetic cell sorting using anti-CD14-coated beads according to manufacturer’s recommendations (MiltenyiBiotec, Auburn, CA) and subsequently cultured for six days in RPMI medium 1640 with 10% FBS and 50 unit/ml recombinant human GM-CSF to generate GM-CSF monocyte-derived macrophage (GM-MDM)[22–25]. IL-4 (25 ng/ml) in RPMI medium 1640 with 10% FBS (adjusted to 300 mOsm) was treated for additional two days to induce DC-SIGN expression. On the 8th day, cells were pre-incubated in 300 mOsm Tyrode's solution for 40 min with 5μg/ml of either goat anti-MRpAb (R&D), or mouse anti-DC-SIGN (R&D), or both and then infected with 0.1 MOI C6/36 derived dengue virus in culture condition for 30 min[5]; then the cell lysate was harvested. Magnetic protein A beads (Taiwan Advanced Nanotech) were cross-linked with rabbit anti-DAP12 pAb (Santa Cruz) by BS3 (Thermo Pierce). DAP12 beads were incubated with cell lysate with protease inhibitor PMSF, and phosphatase inhibitor NaF, and Na_2_VO_3_ for one hr at room temperature according to manufacturer’s recommendation, and then washed, and standard Western blotting was conducted using either mouse anti-Tyr-P mAb (Cell Signaling Technology) or rabbit anti-DAP12 pAb.

### Fluorescence microscopy and transmission electron microscopy

GM-MDM cells were cultured on poly-D-lysine coated coverslips for eight days, with or without IL-4 treatment, and then infected with 0.1 M.O.I. C6/36-derived dengue virus for 5 min in culture condition, and fixed with 4% paraformaldehyde (Electron Microscopy Sciences). Following the standard procedure, the fixed cells were stained with fluoro-dye labeled primary antibodies: goat anti-MR pAb (R&D), rat anti-DC-SIGN mAb (eBiosciences), mouse anti-MDL-1 mAb (R&D), and/or mouse anti-dengue mAb (3H5, EMD Millipore) (Zenon® labeling, Molecular Probes, Life Technologies). After staining, the coverslip was mounted with Fluorescence Mounting Medium (Dako), sealed with clear nail polish, and further analyzed with Zeiss LSM 780. The dengue virus with positive staining was gated to differentiate dengue virus presence and absence areas in dengue virus-infected cells, according to manufacture’s suggestion (Volocity, PerkinElmer). The confocal fluorescence micrographs were further analyzed for localization using the Pearson Coefficient Correlation (PCC) analysis by Volocity (PerkinElmer)[26]. Then the data collections can go further followed by Student's *t*-test or ANOVA test to see if the means of two or several groups are equal.

For transmission electron microscopy (TEM), cells were cultured on the poly-D-lysine coated formvar/carbon film nickel grids (Electron Microscopy Sciences) for eight days, with or without IL-4 treatment. Before fixation, cells were stained with goat anti-MR pAb (R&D), rat anti-DC-SIGN mAb (eBiosciences), mouse anti-MDL-1 mAb (R&D) and the corresponding gold-conjugate 2nd Ab (Electron Microscopy Sciences) at 4°C. Cells were fixed and dried with the standard procedure for TEM (FEI Tecnai G2 F20 S-TWIN) [27–29].

The specificities of the primary anti-dengue virus, anti-mannose receptor, anti-DC-SIGN, and anti-CLEC5A antibodies and their respective saturating doses were established according to manufacturer’s description. Secondary antibodies were used at a 1:10 dilution (A_520_ = 2.0 for antibodies conjugated to 15- or 10-nm gold and 1.0 for antibody conjugated to 5-nm gold). Cells were not labelled with the secondary antibodies when pre-incubated with isotype control antibody or antiserum, or when receptor-specific antibody was omitted.

## Results

### MR and DC-SIGN exhibit much stronger dengue virus binding than CLEC5A

**Fig 1:**
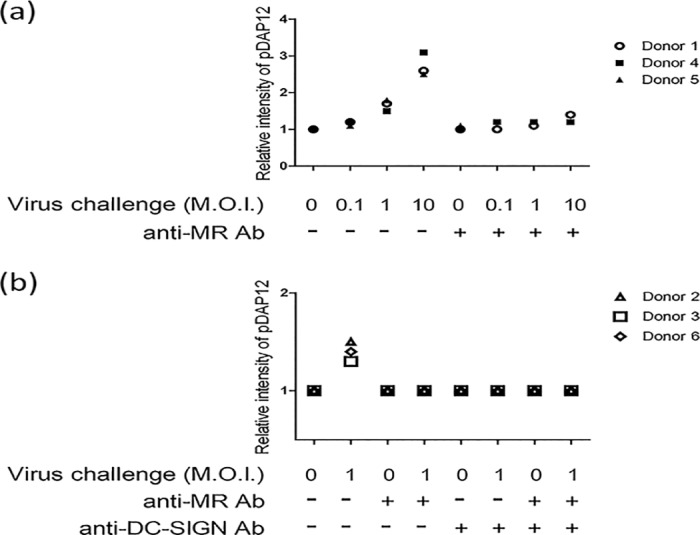
Dengue virus-induced DAP12 Tyr-phosphorylation is mannose binding receptor and DC-SIGN dependent. **(a)** Human GM-MDM cells challenged with 0, 0.1, 1 or 10 MOI dengue virus were treated with anti-mannose receptor (hMR) antibody as a blocker, then immunoprecipitated with phosphor-tyrosine (pY) antibody for quantification of the endogenous DAP12 with Western blot. **(b)** GM-MDM cells were stimulated by IL-4 for 48 hr and then challenged with 1 M.O.I. dengue virus in the presence of the indicated blocker, then the endogenous DAP12 was analyzed with Western blot.

CLEC5A, MR, and DC-SIGN are all able to bind to dengue virus. It is, therefore, reasonable to consider that these three receptors are competitive or independent, or they cooperate with each other in binding to dengue virus. In the study of the binding kinetics between receptors and dengue virus by bio-layer interferometry method, we observed that both MR and DC-SIGN bind the virus very strongly with *K*_D_ in the sub-nanomolar range, but the *K*_D_ between CLEC5A and dengue virus is more than 172 μM, which is more than thousand times weaker than MR and DC-SIGN (Table 1 and S1A and S2B Fig). This result can be further explained as follows: in the presence of CLEC5A and MR/DC-SIGN on the surface of a macrophage, dengue virus particles are captured by the macrophage first through interaction with MR/DC-SIGN instead of CLEC5A, and before MR/DC-SIGN saturate their binding with dengue virus, there will be almost no CLEC5A binding to the virus to transduce signaling. However, in the previously study, we demonstrate that while the normal functions of MR and DC-SIGN are not disturbed, the downstream signaling is CLEC5A dependent[8]. These results indicate that although the binding avidity is very different between CLEC5A and MR/DC-SIGN, CLEC5A still can have sufficient binding to dengue virus to transmit its downstream signaling, suggesting that CLEC5A does not compete with MR/DC-SIGN for dengue virus binding. We further used the anti-MR antibody to block MR on human GM-CSF monocyte-derive macrophage (GM-MDM) cells without DC-SIGN expression (S2 Fig) [22] and found that the CLEC5A-mediated downstream DAP12 tyrosine phosphorylation (DAP12 pY), which is the trigger for downstream cytokine secretion, was downregulated (Fig 1A). We also observed that increasing the dosage of dengue virus challenge resulted in a significant DAP12 phosphorylation, indicating that the CLEC5A/DAP12 signaling pathway was still intact, but not as sensitive as that without anti-MR antibody treatment. Furthermore, treatment of human GM-MDM cells with IL-4 to induce DC-SIGN expression (S2 Fig), followed by addition of either anti-MR antibody or anti-DC-SIGN antibody as a blocking agent also significantly downregulated the DAP12 pY level (Fig 1B). Combining with the binding kinetic study, these results suggest that both DC-SIGN and MR are involved in the virus binding before CLEC5A-mediated DAP12 downstream signaling, and this binding also facilitated CLEC5A/dengue interaction and enhanced CLEC5A-mediated DAP12 downstream signaling.

**Table 1:** Global kinetic analysis of dengue virus binding to different Fc-lectins that immobilized on Octet AHC biosensor.

Immobilized Fc-Lectin (nm)			*k*_on_ (M^-1^s^-1^)	*K*_off_ (s^-1^)	*K*_D_ (nM)^#^
hMR-Fc	Fc-DC-SIGN	Fc-CLEC5A			
0.5	--	--	6.2 (±0.11)x10^6^	3.3 (±0.22)x10^-4^	0.054
--	0.5	--	5.2 (±0.13)x10^6^	1.3 (±0.04)x10^-3^	0.246
--	--	2.0	N/A	N/A	> 172μM*
--	0.5	0.5	2.9 (±0.03)x10^6^	1.2 (±0.09)x10^-3^	0.427
	0.5	2.0	4.9 (±0.03)x10^4^	1.3 (±0.13)x10^-4^	2.560

Bio-layer interferometry (BLI, ForteBio) is used for the kinetic study of human receptor-Fc conjugates interacting with dengue virus. The biosensor (ForteBio) is immobilized with the receptor, and reacts with dengue virus in Tyrode's solution at room temperature. Data were analyzed with 1:1 binding as there is no other proper binding mode to be fitted with this type of multivalent binding. Each *K*_D_ value is the mean (and standard deviation) from two independent experiments.

^*^ *K*_D_ of dengue virus/Fc-CLEC5A is measured by conventional ELISA method.

^#^ The standard deviation of the *K*_D_ value listed in the table are all below 10^−4^ nM.

### Mode of cooperation: multivalent virus-receptor binding complex

**Fig 2:**
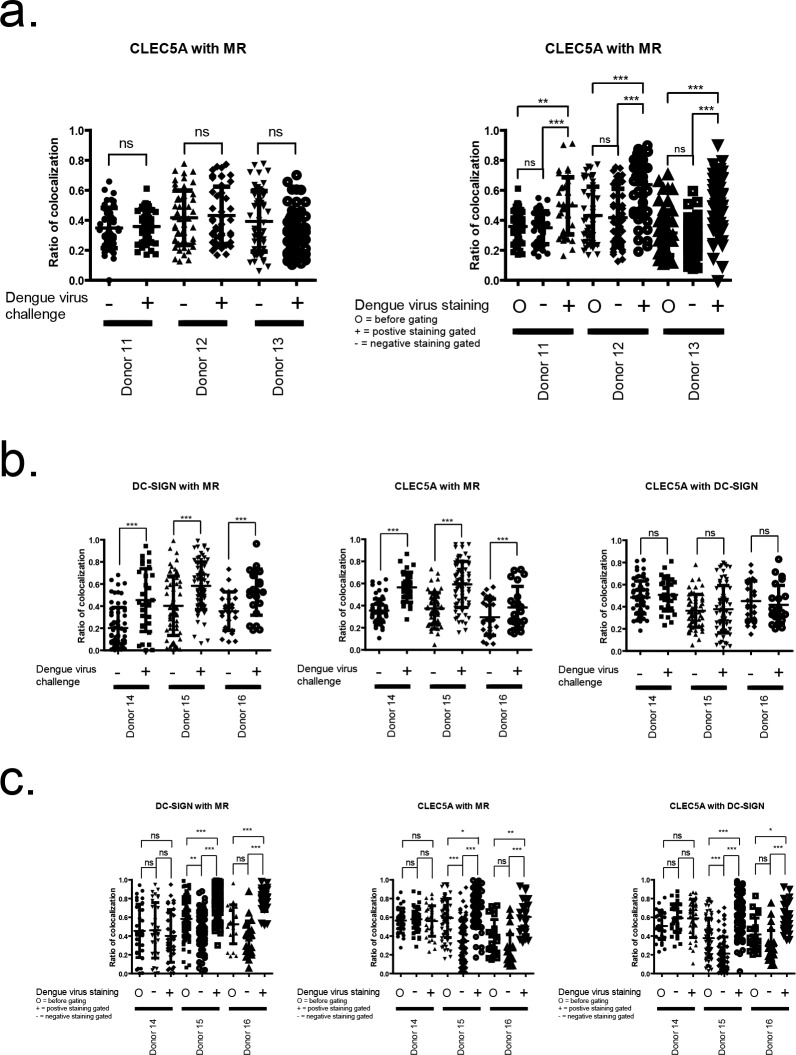
The Pearson Coefficient Correlation (PCC) analysis of co-localization revealed that CLEC5A/MR and CLEC5A/DC-SIGN colocalization is significantly increased in the dengue virus presence area of virus-infected human GM-MDM cells. GM-MDM cells were cultured on coverslips for eight days, with or without IL-4 treatment as described, and then infected with 0.1 M.O.I. C6/36-derived dengue virus. After the cell was fixed and stained, and analyzed by confocal fluorescence microscopy, the confocal fluorescence micrographs were further analyzed for localization using the PCC analysis by Volocity (PerkinElmer). Then the data collections go further followed by Student's *t*-test or ANOVA test to see if the means of two or several groups are equal. Every data point in the figures accounts for a single cell. More than 45 cells are counted in each donor. *p < 0.05, **p < 0.01, ***p < 0.001, ns = no significant. **(a)** PCC analysis of colocalization of CLEC5A with MR in GM-MDM cells in various donors. Comparison of results with or without dengue virus challenge (Left). Comparison of dengue virus gated positive and negative results in dengue virus tested GM-MDM cells (Right). **(b)** PCC analysis of colocalization of DC-SIGN with MR, and CLEC5A with MR, and CLEC5A with DC-SIGN in GM-MDM cells stimulated with IL-4. Various donors are compared with or without dengue virus challenge. **(c)** CC analysis of colocalization of DC-SIGN with MR, and CLEC5A with MR, and CLEC5A with DC-SIGN in GM-MDM + IL-4 stimulated cells challenged with dengue virus. Various donors are compared with dengue virus gated positive and negative cells.

**Fig 3:**
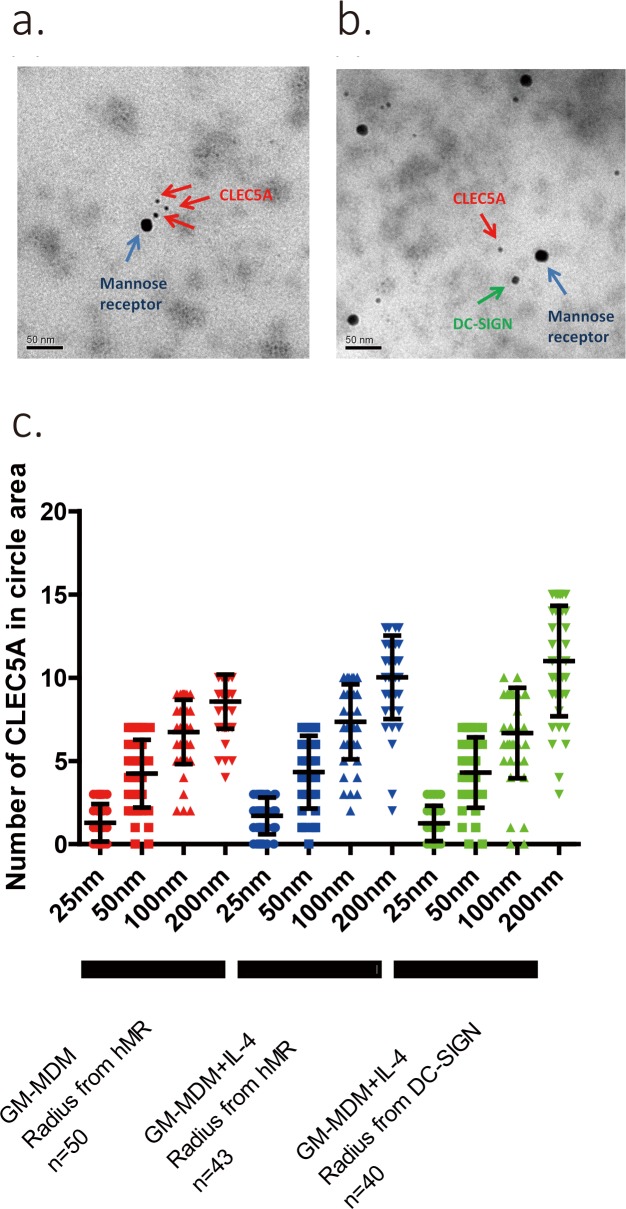
Immunogold assisted TEM revealed close proximity of CLEC5A/MR/DC-SIGN. GM-MDM cells were cultured on grids for eight days, with or without IL-4 treatment. After standard TEM procedure, the image was further analyzed as described. **(a)** Immunogold TEM image of GM-MDM cell membrane with MR (15 nm gold particles), and CLEC5A (5nm gold particles) stained. **(b)** Immunogold TEM image of GM-MDM cell stimulated by IL-4; the membrane was stained with anti-MR (15 nm gold particles), anti-DC-SIGN (10 nm gold particles) and anti-CLEC5A (5 nm gold particles) antibodies. **(c)** Statistical result of the number of CLEC5A-immunogold dots counted within various radius ranges using MR/DC-SIGN as the centre of a circle.

The enhancement of CLEC5A/DAP12 signaling by MR/DC-SIGN in the dengue virus challenging study suggests the importance of virus-host cell binding in the host innate immune response. It also suggests that the binding between the virus and MR/DC-SIGN and the virus and CLEC5A might occur in a cooperative mode. But, it is unclear how the functional cooperation between CLEC5A and MR/DC-SIGN on the surface of human macrophage is established. We first analyze the possible colocalization between CLEC5A and MR or CLEC5A and DC-SIGN, with and without dengue virus challenge (S3 Fig). Analysis of PCC revealed that the CLEC5A/MR and CLEC5A/DC-SIGN colocalization correlation is significantly increased in the area of virus-infected human GM-MDM cells (Fig 2). We further look into the CLEC5A/MR/DC-SIGN colocalization area by TEM with specific immunogold labeling which provides higher resolution than conventional confocal microscopy (Fig 3A and 3B). From the results, we know that after the challenge with dengue virus, the spatial colocalization of MR/CLEC5A on GM-MDM does not change. We use MR/DC-SIGN as centre point, and define a specific radius for the circle area: 25 nm radius circle represents a single virus projection area, 50 nm radius circle represents minimal space necessary for the whole embedded virus particle, 100nm radius circle represents 4-virus projection area, and 200nm radius circle represents multi-virus projection zone (S4 Fig). By counting the number of CLEC5A immunogold dots in a specific area, we found that in the CLEC5A/MR/DC-SIGN colocalized area, a single MR/DC-SIGN is surrounded by multiple CLEC5A immunogold dots, which increase in number with increase in the radius of the circle (Fig 3C). This data also provide direct evidence about the formation of hetero-multivalent virus-receptor binding complex.

The receptor-virus complex surrounded by a low-affinity receptor was further studied kinetically. We immobilized DC-SIGN/CLEC5A as a 1/4 ratio on the tip of BLI, and found that when comparing to DC-SIGN as high-affinity receptor alone, the overall *K*_on_ decreased by two orders of magnitude, and *K*_off_ decreases by one order of magnitude, resulting in a one order of magnitude decrease in *K*_D_ (Table 1 and S1C and S1D Fig). In contrast, this high/low-affinity receptor mixture increases the overall binding equilibrium by more than three orders of magnitude comparing to CLEC5A alone. This result also demonstrates a plausible mechanism that the weak affinity CLEC5A is in close proximity to the high-affinity receptor to form a multivalent virus-receptor binding complex and facilitate CLEC5A signal transduction.

## Discussion

The hetero-multivalent virus-receptor binding complex proposed in this study provides evidence that the host defense against pathogen invasion does not require an all-purpose receptor that binds the pathogen and transmits signals independently without the involvement of other receptors. This model supports the hypothesis that macrophage cells achieve efficient pathogen binding and stimulate signal transduction through a different site, which is in close proximity to the primary binding receptor.

Before this study, the binding of dengue virus to the binding receptor (MR/DC-SIGN) and the binding of the virus to the signal receptor CLEC5A are two independent events (S5A Fig)[14]. But, this kind of monovalent binding cannot explain the result of our study, i.e. binding receptors are functionally correlated with the signaling receptor and both events occur concurrently. Logically, only the model of multivalent hetero-complex can have a good fit with our discovery (S5B Fig). So, combined with our co-localization correlation study and kinetic study above, we conclude that this virus-receptor hetero-complex should be the initiator of CLEC5A-mediated signal transduction. Our kinetic study also reveals that in the pathway to form DC-SIGN-DV-CLEC5A complex, DC-SIGN and CLEC5A are cooperative in binding to the virus, and as the more CLEC5A in the DC-SIGN-DV-CLEC5A virus-receptor multivalent hetero-complex, the weaker its *K*_D_ is.

**Fig 4:**
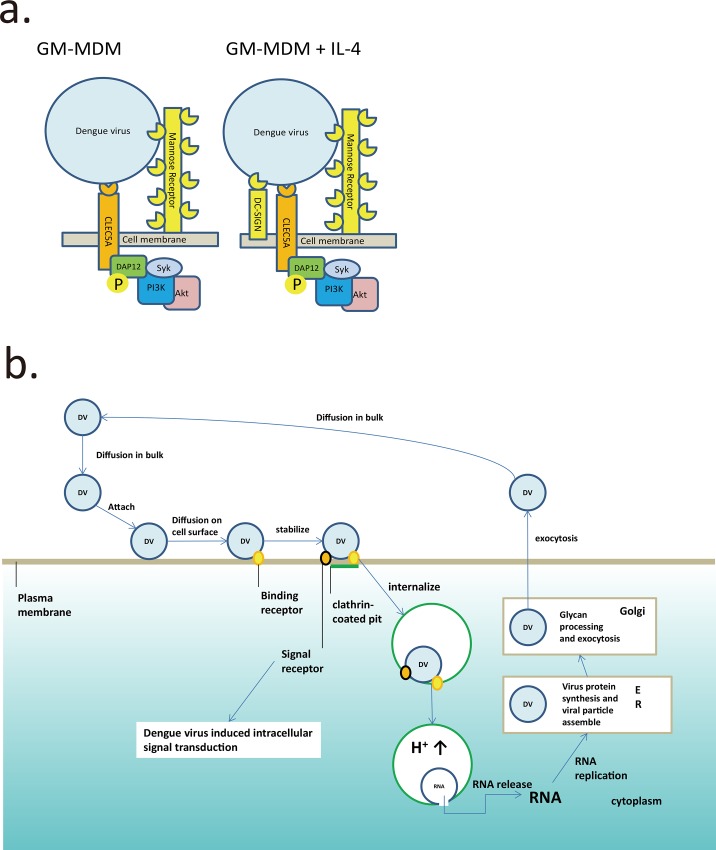
Dengue virus infection is through a cooperative interaction between a mannose receptor and CLEC5A on macrophage as a multivalent hetero-complex. **(a)** Binding model of dengue virus with MR/DC-SIGN and CLEC5A on GM-MDM cell membrane. **(b)** The strong avidity receptor captures dengue virus to facilitate the interaction with a weak-binding signal receptor in proximity to induce innate immune response.

It is known that after the dengue virus particle attaches to the cell surface and is stabilized, its diffusion constant at 37°C is less than 0. 025μm^2^/sec [4]. Considering the practical diffraction limit of the conventional microscope (0.2 μm), we can assume that the stabilized virus does not move under the microscope conditions. Furthermore, after endocytosis, no more dengue virus binding will occur outside the endocytosis vesicle. Therefore, if the dengue virus binding to these receptors is not within the same binding area or space, there should be no cooperation. Therefore, taking our co-localization correlation analysis, TEM image analysis, and binding kinetic study, we propose that CLEC5A and MR/DC-SIGN are located in close proximity on the cell surface of the macrophage for an efficient innate immune response (Fig 4A). In this model, we assume that the dengue virus particle has a diameter of about 50 nm and the projection area of the virus is also around 50 nm in diameter. For an active cooperation between these two receptors, both should be localized in this projection area. However, we did not exclude the possibility that the cooperation of these receptors may not be in this projection area. After one of the receptors is bound to the virus, the other receptor could be recruited to the proximity. Alternatively, the virus could exist as a multi-particle cluster to form the multivalent complex in the projection area. All these results further demonstrate that the binding between dengue virus and MR, DC-SIGN, and CLEC5A occurs on the cell surface as a hetero-multivalent complex that enhances human macrophage CLEC5A/DAP12 signaling.

In the kinetic study, although we had difficulty to measure the *K*_on_ value for CLEC5A binding to dengue virus due to the very weak association, the difference in dengue virus association between CLEC5A and MR/DC-SIGN is significant. Because of the high avidity between dengue virus and MR/DC-SIGN, it should not be possible for CLEC5A to bind to the virus independently, especially when MR and DC-SIGN are much larger molecules than CLEC5A, and would not favor prior binding of CLEC5A spatially. For these reasons, dengue virus may bind to MR/DC-SIGN first as the primary receptor, followed by a concurrent interaction with CLEC5A as the signal co-receptor in close proximity (Fig 4B). But whether the binding and signaling stimulation are sequential or in parallel needs further study. Furthermore, it has been shown by us and others that the mosquito-derived dengue virus contains high-mannose type glycoforms and the initial receptor for the virus to infect human cells is mannose binding receptor, and the following human cell-derived dengue viruses contain complex type N-glycoforms which selectively interact with DC-SIGN[5, 18, 19, 30, 31]. This kind of binding receptor switch further highlight the cooperativity between the binding receptor and signal receptor and the cooperativity provides better defense efficiency.

The carbohydrate binding domain (CRD) of DC-SIGN has been shown to bind to specific envelope protein pairs on dengue virus, suggesting that DC-SIGN and dengue virus-CRD interaction is involved in a high order structure[32]. In the TEM results, we also found that MR /DC-SIGN/CLEC5A may bind to different portions of the dengue virus, which means that they do not bind to the same E protein. This assumption is based on the TEM image results without virus attaching to the receptors. The binding mode and dynamics of the CLEC5A/MR/DC-SIGN interaction are obviously much more complex when compared to DC-SIGN-dengue virus interaction alone, and a new method may be developed to elucidate the details of this multivalent interaction.

In response to dengue virus infection, the GM-MDM cells use CLEC5A as an innate immune receptor, and this CLEC5A/DAP12 innate immune response is facilitated by the high avidity glycan binding receptors MR and DC-SIGN in proximity to form a hetero-multivalent complex. However, our data represent a steady state result, and the real dynamics of cell surface binding remain unclear. Though attempts to elucidate the entry pathway has been reported, a single-molecule imaging method may be required to understand the molecular mechanism of dengue virus infection.[33]

## Supporting Information

S1 Fig Bio-layer interferometry kinetics study of human Fc-receptor and virus binding. Bio-layer interferometry (BLI, ForteBio) is used for the kinetic study of human receptor-Fc conjugates interacting with dengue virus. The biosensor (ForteBio) is immobilized with the receptor and reacts with dengue virus in Tyrode's solution at room temperature. The concentration of dengue virus in the study was determined based on the equivalent of dengue virus envelope protein. Data were analyzed with 1:1 binding as there is no other proper binding model to be fitted with this type of multivalent binding. **(a)** Kinetic study of MR-Fc binding to dengue virus. **(b)** Kinetic study of Fc-DC-SIGN binding to dengue virus. **(c)** Fc-DC-SIGN and Fc-CLEC5A are sequentially immobilized on detection probe as 0.5nm/0.5nm ratio, and kinetic study was performed with dengue virus binding. **(d)** Fc-DC-SIGN and Fc-CLEC5A are sequentially immobilized on detection probe as 0.5nm/2.0nm ratio, and kinetic study was performed with dengue virus binding.
(TIF)

S2 Fig Differential expressions of MR, DC-SIGN and CLEC5A in GM-MDM w/o IL-4 stimulation. GM-MDM w/o IL-4 stimulation was generated as described and subsequently analyzed by flow cytometry for surface receptor expression and normalized with isotype control. Bars represent the SD of at least 3 independent experiments. One donor is shown.
(TIF)

S3 Fig Confocal fluorescence micrographs of human macrophage challenged with dengue virus. Staining of either dengue virus, or DC-SIGN, or MR, or CLEC5A as indicated. Images showing the colocalized pixels of DC-SIGN with MR, and CLEC5A with MR, and CLEC5A with DC-SIGN are also presented in false color. The dengue virus positive staining is gated to differentiate dengue virus presence and absence areas in dengue virus infected cells. **(a)** Confocal fluorescence micrographs of human GM-MDM cells with indicated staining. **(b)** Confocal fluorescence micrographs of human GM-MDM cells + IL-4 stimulation with indicated staining.
(TIF)

S4 Fig Calculation of radius circle for MR/DC-SIGN with CLEC5A. We use MR/DC-SIGN as centre point, define specific radius for circle area: 25nm radius circle represents a single virus projection area, 50nm radius circle represents minimal area necessary for the whole embedded virus particle, 100nm radius circle represents 4- virus projection area, and 200nm radius circle represents multi-virus cluster projection areas.
(TIF)

S5 Fig The multivalent binding model provides an efficient way of function through a cooperation between the binding receptor and the signaling receptor. **(a)** The monovalent binding model cannot explain the function through the cooperation between binding receptor DC-SIGN and signaling receptor CLEC5A. **(b)** The multivalent binding model provides a better fit of current observation of functional and binding cooperation.
(TIF)
