# Supplementary figures and images for: Dengue Virus Infection Is through a Cooperative Interaction between a Mannose Receptor and CLEC5A on Macrophage as a Multivalent Hetero-Complex
Source: PLoS One. 2016 Nov 10;11(11):e0166474. doi: 10.1371/journal.pone.0166474 (PMC5104462; doi:10.1371/journal.pone.0166474)

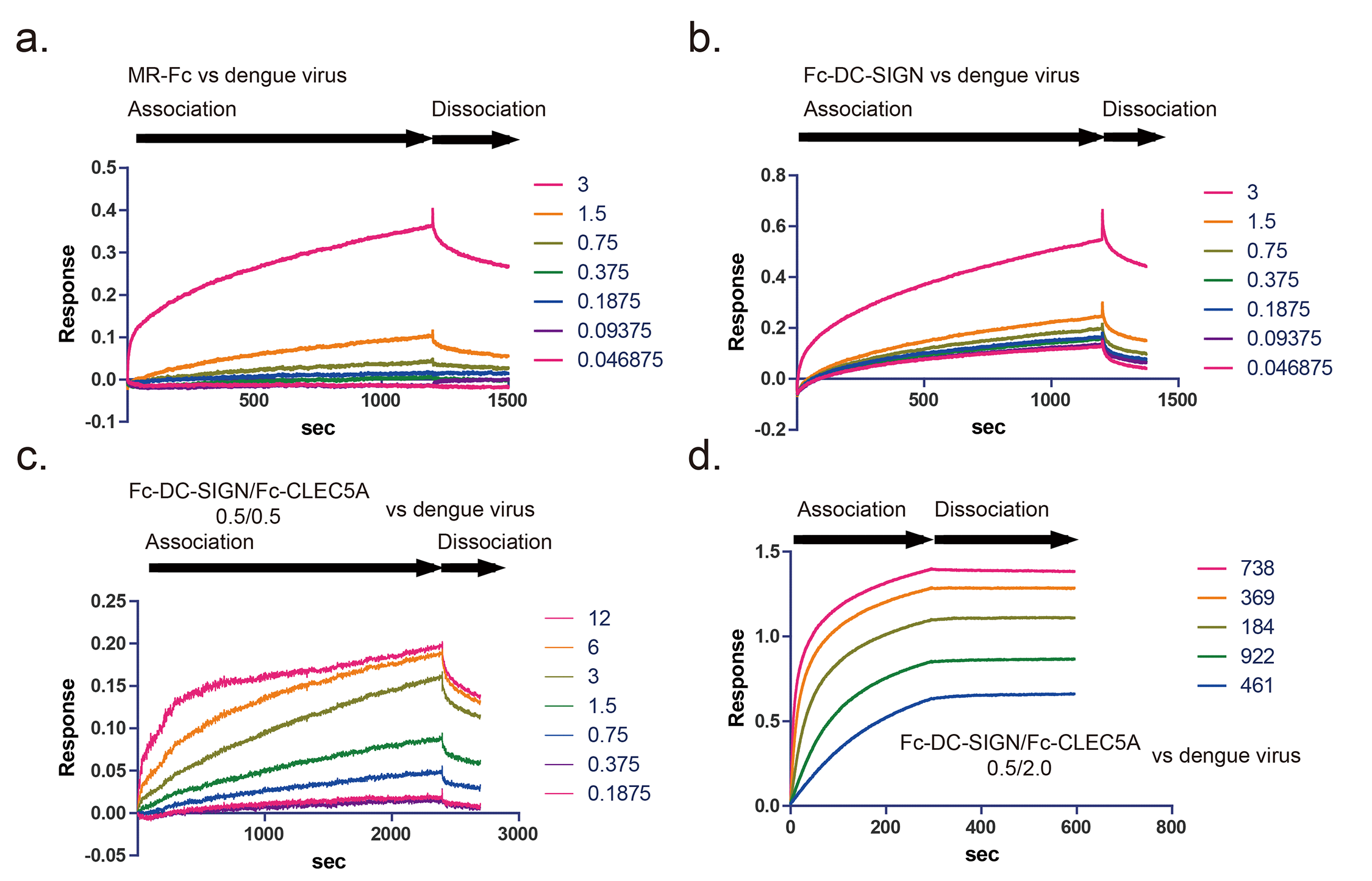

Supplement: S1 Fig — Bio-layer interferometry (BLI, ForteBio) is used for the kinetic study of human receptor-Fc conjugates interacting with dengue virus. The biosensor (ForteBio) is immobilized with the receptor and reacts with dengue virus in Tyrode's solution at room temperature. The concentration of dengue virus in the study was determined based on the equivalent of dengue virus envelope protein. Data were analyzed with 1:1 binding as there is no other proper binding model to be fitted with this type of multivalent binding. (a) Kinetic study of MR-Fc binding to dengue virus. (b) Kinetic study of Fc-DC-SIGN binding to dengue virus. (c) Fc-DC-SIGN and Fc-CLEC5A are sequentially immobilized on detection probe as 0.5nm/0.5nm ratio, and kinetic study was performed with dengue virus binding. (d) Fc-DC-SIGN and Fc-CLEC5A are sequentially immobilized on detection probe as 0.5nm/2.0nm ratio, and kinetic study was performed with dengue virus binding. (TIF) [file pone.0166474.s001.tif]

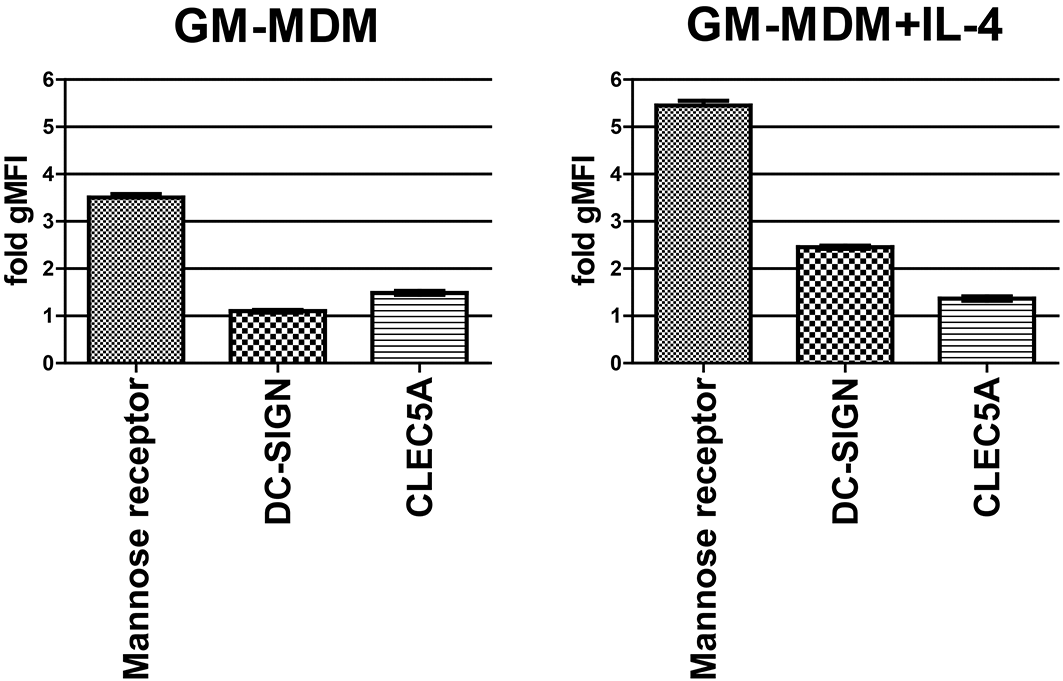

Supplement: S2 Fig — GM-MDM w/o IL-4 stimulation was generated as described and subsequently analyzed by flow cytometry for surface receptor expression and normalized with isotype control. Bars represent the SD of at least 3 independent experiments. One donor is shown. (TIF) [file pone.0166474.s002.tif]

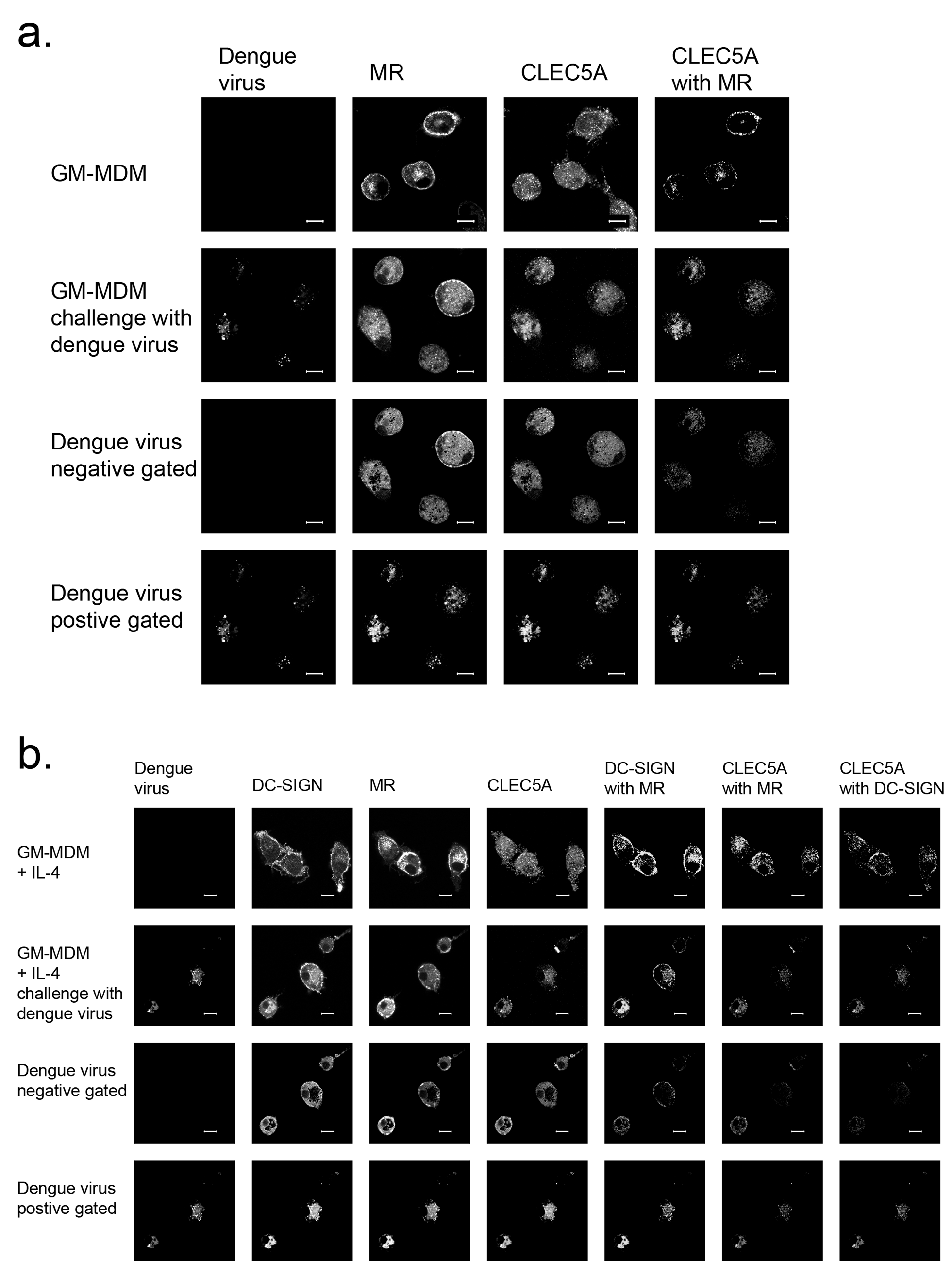

Supplement: S3 Fig — Staining of either dengue virus, or DC-SIGN, or MR, or CLEC5A as indicated. Images showing the colocalized pixels of DC-SIGN with MR, and CLEC5A with MR, and CLEC5A with DC-SIGN are also presented in false color. The dengue virus positive staining is gated to differentiate dengue virus presence and absence areas in dengue virus infected cells. (a) Confocal fluorescence micrographs of human GM-MDM cells with indicated staining. (b) Confocal fluorescence micrographs of human GM-MDM cells + IL-4 stimulation with indicated staining. (TIF) [file pone.0166474.s003.tif]

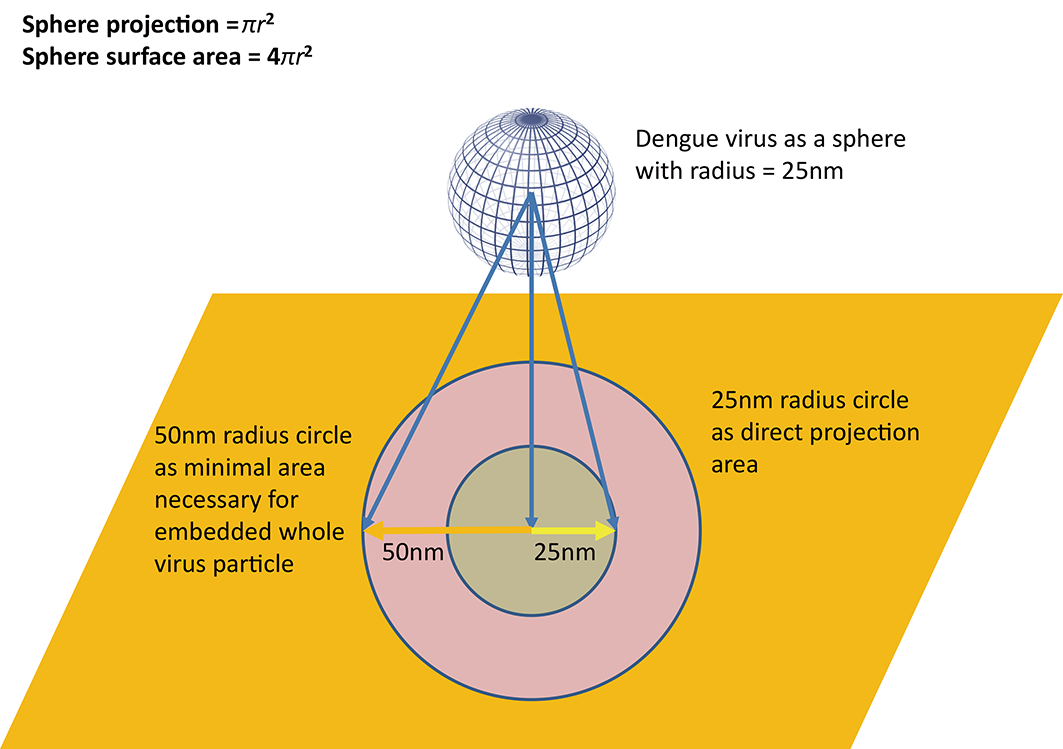

Supplement: S4 Fig — We use MR/DC-SIGN as centre point, define specific radius for circle area: 25nm radius circle represents a single virus projection area, 50nm radius circle represents minimal area necessary for the whole embedded virus particle, 100nm radius circle represents 4- virus projection area, and 200nm radius circle represents multi-virus cluster projection areas. (TIF) [file pone.0166474.s004.tif]
